# The Associative Structure of Memory for Multi-Element Events

**DOI:** 10.1037/a0033626

**Published:** 2013-08-05

**Authors:** Aidan J. Horner, Neil Burgess

**Affiliations:** 1 Institute of Cognitive Neuroscience and Institute of Neurology, University College London, London, England

**Keywords:** episodic memory, hippocampus, source memory, multimodal binding, pattern completion

## Abstract

The hippocampus is thought to be an associative memory “convergence zone,” binding together the multimodal elements of an experienced event into a single engram. This predicts a degree of dependency between the retrieval of the different elements comprising an event. We present data from a series of studies designed to address this prediction. Participants vividly imagined a series of person–location–object events, and memory for these events was assessed across multiple trials of cued retrieval. Consistent with the prediction, a significant level of dependency was found between the retrieval of different elements from the same event. Furthermore, the level of dependency was sensitive both to retrieval task, with higher dependency during cued recall than cued recognition, and to subjective confidence. We propose a simple model, in which events are stored as multiple pairwise associations between individual event elements, and dependency is captured by a common factor that varies across events. This factor may relate to between-events modulation of the strength of encoding, or to a process of within-event “pattern completion” at retrieval. The model predicts the quantitative pattern of dependency in the data when changes in the level of guessing with retrieval task and confidence are taken into account. Thus, we find direct behavioral support for the idea that memory for complex multimodal events depends on the pairwise associations of their constituent elements and that retrieval of the various elements corresponding to the same event reflects a common factor that varies from event to event.

Episodic memories are composed of multiple elements, from the people we meet, to the objects we interact with, to the locations we navigate through. Despite this multimodal information being represented in separate neocortical regions, we are seemingly able to retrieve distinct memories that reflect the rich multimodal nature of the original event. The hippocampus, a region critical to episodic memory (Cohen & Eichenbaum, 1993; Kinsbourne & Wood, 1975; O’Keefe & Nadel, 1978; Scoville & Milner, 1957; Squire & Zola-Morgan, 1991), is thought to underlie this ability—acting as a “convergence zone” to bind multimodal information into a single engram (Damasio, 1989; Marr, 1971; McClelland, McNaughton, & O’Reilly, 1995; Teyler & DiScenna, 1986). This proposal fits with the idea that episodic memory is supported by the existence of event engrams, discrete bound representations containing information about multiple event elements (Tulving, 1983).

Despite this hypothesis from both neuroscientific and psychological theories of episodic memory, little evidence has been presented relating to the associative structure of the representation of multi-element events. If event engrams correspond to the association of multiple elements (e.g., locations, people and objects) to form a single representation, there should be some degree of dependency between the retrieval of elements from the same event compared with the retrieval of elements from different events. Thus, the probability of successfully retrieving one element from an event should be related to the probability of successfully retrieving another element from the same event. Alternatively, pairwise associations between different within-event elements could be encoded and retrieved independently, predicting no relationship between the retrieval of two different elements from the same event. To date, research into episodic memory has focused on memory either for lists of single items or for associations between pairs of items or between items and their “source” or “context.” Although the hippocampus has been implicated in supporting these associations, and specifically cross-modal associations (e.g., Eichenbaum, Yonelinas, & Ranganath, 2007; Hannula, Tranel, & Cohen, 2006; Mayes, Montaldi, & Migo, 2007; Vargha-Khadem et al., 1997), previous studies did not address the issue of dependency across multiple elements within the same event.

To test the hypothesis that cued retrievals of the elements comprising a single event are related, events with more than two elements need to be tested. In the present study, we required participants to learn location–person–object triplets. Using three elements, we can ask how dependent the retrieval of one element (e.g., the person) is on retrieval of another element (e.g., the object) when cued by the third element (e.g., the location). Previous research has focused on the sequential pairwise associative relationships among three distinct elements. For example, by asking how the existence of a pre-existing association (e.g., A–to–B) affects the encoding of a new overlapping association (e.g., A–to–C; Martin, 1971), how the retrieval of noncued associated elements (e.g., A–to–B) when learning a new association (e.g., A–to–C) promotes the encoding of multi-element bindings (e.g., encoding of B–to–C; Zeithamova, Dominick, & Preston, 2012) or how the repeated retrieval of a learned association (e.g., A–to–B) decreases the probability of retrieving other previously associated elements (e.g., A–to–C; retrieval-induced forgetting, or RIF; Anderson, Bjork, & Bjork, 1994). Given our focus on episodic event memory, however, we focused on the relationship among three elements when encoded simultaneously, a situation more akin to the encoding of episodic events in the real world.

Jones (1976) tested memory for more than two elements, requiring participants to learn object–color–location events shown as a two-dimensional photograph. At test, participants were cued with either one or two elements and were required to recall the remaining element(s). Although performance improved when two cues were presented relative to one, the improvement was less than expected if the two cues were independently associated with the tested element. Jones proposed a *fragmentation hypothesis* where mnemonic representations could contain any or all elements within an event. The proposal of an engram that contains all elements of an event accords with Tulving’s (1983) idea of a single bound event. These studies however did not assess the dependency of retrieving elements within an event. Stronger evidence in favor of a bound event would entail showing that on an event-by-event basis, the probability of retrieving one element was contingent on the probability of retrieving another.

Within the source memory literature, episodic dependence has been assessed by associating single items (e.g., a word) with two “sources” (e.g., location and font type). For example, Meiser and Bröder (2002) provided evidence for a degree of dependence in retrieving both source elements when presented with the item, particularly in situations associated with the phenomenological experience of recollection (Gardiner, 1988). More recently, however, it has been suggested that source details are principally associated with the item and not with each other (Starns & Hicks, 2005, 2008), suggesting a degree of asymmetry in relation to object–color–location representations whereby color source information and location source information are independently bound to object information but not to each other (also see the idea of *headed records*; Morton, Hammersley, & Bekerian, 1985). Such source memory experiments combine a single item with multiple sources, giving a primacy to a single element within an event. Furthermore, using object features as source details (e.g., color) may not require the multimodal binding mechanisms the hippocampus is thought to perform. We were interested in examining dependency among distinct event elements that are likely individually represented within different neocortical regions. Furthermore, each element was an individual “item” in the sense that no element was a subordinate feature of another element (e.g., the color of an object).

We previously showed that cued retrieval of the elements of events experienced in a virtual reality computer game, composed of an object, a place, and either a person or an odor, was impaired by hippocampal damage (Spiers, Burgess, Hartley, Vargha-Khadem, & O’Keefe, 2001; Spiers, Burgess, Maguire, et al., 2001; see also Burgess, Maguire, & O’Keefe, 2002). In a speculative analysis of these data (Trinkler, King, Spiers, & Burgess, 2006), we failed to find any significant dependence between the cued retrieval of different elements of the same event. However the small numbers of events, low performance levels in the paired forced-choice memory tests, and unsophisticated analyses severely reduced the power of this analysis, making the result ambiguous.

In the present experiments, in order to engage the hippocampal system thought to underlie episodic binding, we presented participants with three distinct event elements (as words)—a location, famous person, and common object (e.g., a supermarket, Barack Obama, and a pencil case; see also Yim, Dennis, & Sloutsky, 2011). They were instructed to construct a scene involving all three elements and imagine themselves “as vividly as possible” within the constructed scene. For example, they might imagine Barack Obama at a supermarket checkout buying a pencil case. This scene construction encoding task was again designed to engage the hippocampus (see Addis, Wong, & Schacter, 2007; Byrne, Becker, & Burgess, 2007; Hassabis, Kumaran, & Maguire, 2007; Hassabis, Kumaran, Vann, & Maguire, 2007) to a greater extent than previous multi-element source memory studies. Finally, as we were principally interested in the encoding of bound events, we tested memory for these events across multiple retrieval trials by cuing with a single element and testing either one (Experiments 2–3) or both (Experiment 1) uncued elements. Thus, we could examine how the probability of retrieving one element depended on the probability of retrieving another element of the same event.

Using this experimental paradigm, we assessed episodic dependency across three experiments. First, we created contingency tables based on performance for two elements from the same event when cued by the remaining element (as well as performance for a single element when cued by the two remaining elements across retrieval trials). For instance, we constructed a 2 × 2 contingency table based on the probability of retrieving the person and object when cued by location. The existence of discrete event engrams would predict a degree of dependency such that most retrieval trials should either successfully or unsuccessfully retrieve both the person and object. In other words, retrieving the person but not the object (and vice versa) should be rare. To appropriately assess dependency, controlling for overall memory performance, we created an independent model that assumes independent pairwise associations are formed among the elements composing an event and that cued retrieval of one element by another element reflects the strength of the corresponding association. The independent model served as a baseline (i.e., the null hypothesis) against which we compared the degree of dependency in the data across three experiments. These experiments provide strong evidence for episodic dependency, and therefore, we built further models designed to account for such evidence. First, in Experiment 1, using a cued-recall task at retrieval we provide strong evidence against the independent model.

## Experiment 1

### Method

#### Participants

Fourteen participants (five men) gave informed consent to participate in Experiment 1. One participant was excluded from further analyses due to 100% cued-recall performance. The remaining 13 participants had a mean age of 24.2 years (*SD* = 3.9). By self-report, all participants were right-handed. All experiments were approved by the University College London Research Ethics Committee (NB/PWB/26102011a).

#### Materials

Stimuli were 24 locations (e.g., a swimming pool), famous personalities (e.g., David Cameron), and common objects (e.g., a bicycle; see the Appendix for a full list of stimuli). Four randomized sets of events (i.e., location–person–object triplets) were created. For each of the four sets, a pseudo-randomized test order was generated (details provided later). These four sets were rotated across participants to reduce possible stimulus or test-order effects.

#### Procedure

The experiment consisted of a single study and test phase. At study, participants were serially presented with all 24 events, each consisting of a location, a famous person, and an object. All three items within an event were presented on a computer screen as words. The location was always presented in the middle of the screen just above fixation, with the person appearing toward the bottom left and the object toward the bottom right. The items remained onscreen for 12 s. During this time, participants were required to imagine an event that contained all three items. They were told to be “as imaginative as possible” in combining the three items and imagine the event “as vividly as possible.” Each event was preceded by a 3-s fixation cross. The order of event presentation was randomized across participants.

At test, participants performed a cued-recall task. Each trial consisted of a single item from an event (e.g., a location), and participants were required to recall the other two items from the same event (e.g., the person and object). They were also required to give a confidence rating ranging from 1 (*low confidence*) to 5 (*high confidence*) for each of the recalled items. Participants were instructed to give a low confidence if they thought they might have retrieved the correct item; however, they were told to report “don’t know” instead of guessing (confidence was not recorded for these trials). Participants verbally reported the recalled items and confidence ratings. The experimenter recorded the accuracy and confidence for each trial on a preprinted answer sheet that contained the correct answers. A cued-recall answer was only deemed correct if the exact name used at encoding was used (i.e., phonetically or semantically similar answers were marked as incorrect). The experimenter was silent during this period until an answer was given. If the participant failed to report an answer within 30 s of the cue being presented, the experimenter marked the trial as incorrect, and the next cue was presented.

Recall of each event was separately cued using each of the three items (location, person, and object), making a total of 72 cued-recall trials. All 24 events were tested across three blocks. In each block, eight events were cued by location, eight by person, and eight by object. The two subsequent blocks cued each event using the remaining noncued items (e.g., if cued by location in the first block, the second block may have been cued by person and the third by object). Within this structure, four pseudo-randomized test sequences were created and rotated across participants. For each test sequence, the order of cue type across blocks for each event and the order of testing each event within each block were randomized. Prior to the main experiment, participants were given a single event, with three items not used in the main experiment, to practice both the encoding task and the cued-recall task.

#### The independent model

In order to assess the dependency of memory performance for items within individual events, we created an independent model of cued-recall performance (and cued-recognition performance; see Experiments 2 and 3). The contingency table for the independent model is presented in Table 1. The contingency table shows how the probability of successfully or unsuccessfully retrieving a single item within an event depends on the probability of retrieving the other item. Each event (*i* = 1 to *N*) is composed of three items, A (location), B (person), and C (object). For any given participant, the proportion of correct retrievals of B (over *N* events) when cued by A is denoted by *P_AB_* (i.e., the mean performance across all events for B when cued by A). For the independent model, when cued by A, the probability of (a) correctly retrieving both B and C (for all events) is equal to *P*_AB_*P_*AC*_; (b) correctly retrieving B but not C is equal to *P*_AB_*(1 − *P*_*AC*_); (c) correctly retrieving C but not B is equal to (1 − *P*_*AB*_)*P_*AC*_; and (d) incorrectly retrieving B and C is equal to (1 − *P*_*AB*_)*(1 − *P*_*AC*_).[Table-anchor tbl1]

Contingency tables for the observed data and the independent model were created for each individual participant for each cue type to assess the dependency between the retrieval of two items (e.g., person and object) using the same cue type (e.g., location) within a single cued-recall trial (the A_B_A_C_ analysis). We also assessed the degree of dependency for retrieving an individual item (e.g., location) when cued using different items (e.g., person vs. object), across two cued-recall trials (the B_A_C_A_ analysis; see also Trinkler et al., 2006). Thus, six contingency tables were created for each participant for the observed data and the independent model. For the observed data, each cell within a 2 × 2 contingency table contains the number of events whose retrieval resulted in the outcome corresponding to that cell (e.g., both B and C retrieved correctly when cued by A for the top-left cell in the A_B_A_C_ analysis). For the independent model, each cell contains the number expected from an independent combination of the overall performance levels for each pairwise association (e.g., *P*_AB_*P_AC_*N).

#### Dependency measure

To relate the data to the independent model, we calculated a measure of dependency (D) by summing the leading diagonal cells for each 2 × 2 contingency table and dividing this by the overall number of events (known as *accuracy* within binary classification tests). This measure therefore reflects the proportion of trials in which both items comprising an event were either successfully or unsuccessfully retrieved, where 1 = *full dependence* and 0.5 = *full independence* (a score of < 0.5 reflects dependency in the opposite direction such that successful retrieval of one item is less likely, given successful retrieval of another item within the same event).

Using this dependency measure, we first performed 2 × 3 (analysis type [A_B_A_C_ vs. B_A_C_A_] × item type [location vs. person vs. object]) analyses of variance (ANOVAs), comparing dependency for the data across analysis type and item type. Note that the item type (location, person, or object) for the A_B_A_C_ analysis relates to the cue type, whereas for the B_A_C_A_ analysis, it relates to the retrieval type. Given that we saw no consistent differences in dependency across these factors across experiments (see Results), we averaged across them and compared dependency in the data to the independent model. Note that the raw dependency measure is dependent on overall performance—the higher the performance, the greater the dependency in the data and the independent model—therefore, comparisons between the data and the independent model are used to assess this raw measure. Indeed, the independent model is explicitly designed to assess dependency without the confounding presence of accuracy (see Brady, Konkle, Alvarez, & Oliva, 2012, for discussion of this issue).

### Results

#### Cued-recall performance

Performance was well above chance (which would be ∼4%, given the 24 possible responses to each cued-recall trial) with performance at 57% across cue type and retrieval type (see Table 2). Performance across cue type and retrieval type appeared well matched, ranging from 55% to 61%. A one-way within-subject ANOVA across retrieval type (collapsed across cue type) revealed a trend for a main effect, *F*(1.7, 20.0) = 3.11, *p* = .08, η_p_^2^ = .21, with poorer performance for people than for objects, *t*(12) = 3.24, *p* < .01, *d* = 0.52, remaining contrasts: *t*(12)s < 1.2, *p*s > .28. A similar ANOVA across cue type (collapsed across retrieval type) failed to reveal a main effect, *F*(1.8, 22.0) = .46, *p* = .62, η_p_^2^ = .04. Cued-recall performance was therefore poorer for people than for objects; however, there was little evidence for differences across cue type. Finally, a one-way ANOVA revealed that cued-recall performance differed across subjective confidence, *F*(1.8, 11.1) = 16.96, *p* < .001, η_p_^2^ = .74, with an increase in performance from lowest confidence (15.8% accuracy) to highest confidence (95.9% accuracy).[Table-anchor tbl2]

#### Dependency analysis

The mean proportion of trials in which both elements were either successfully or unsuccessfully retrieved across participants (i.e., the dependency measure) for each A_B_A_C_ and B_A_C_A_ analysis and each item type for the observed data are shown in Table 3. We first performed a 2 × 3 (analysis type [A_B_A_C_ vs. B_A_C_A_] × item type [location vs. person vs. object]) ANOVA to ensure the dependency measure was consistent across these factors. Unexpectedly, we saw a significant main effect of analysis type, *F*(1, 12) = 6.96, *p* < .05, η_p_^2^ = .37, with greater dependency in the A_B_A_C_ than B_A_C_A_ analysis. This effect may have been due to the A_B_A_C_ analysis assessing dependency within retrieval trials, whereas the B_A_C_A_ analysis assessed dependency across retrieval trials. We also saw a main effect of item type, *F*(1.9, 22.5) = 3.83, *p* < .05, η_p_^2^ = .24, but no significant interaction, *F*(1.8, 22.1) = 0.33, *p* = .70, η_p_^2^ = .03. Given this latter main effect was not seen consistently across Experiments 1–3, it is not discussed further. We subsequently averaged across these conditions for the data and the independent model prior to model comparison.[Table-anchor tbl3]

**Figure 1 fig1:**
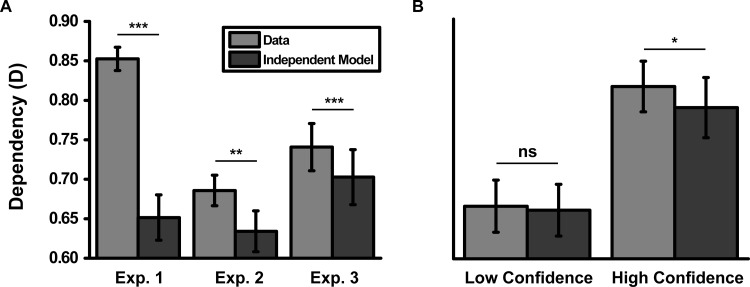
Mean dependency (D) for the observed data and independent model, collapsed across the A_B_A_C_ and B_A_C_A_ analyses and item type, for (A) Experiments 1–3 and (B) low and high confidence events for Experiment 3. Error bars represent ±1 standard error. Exp. = experiment; *ns* = nonsignificant. * *p* < .05. ** *p* < .01. *** *p* < .001.

Dependency (D) for the data and independent model is shown in Figure 1. Comparing the data to the independent model, we found significantly greater dependence than the independent model, *t*(12) = 8.58, *p* < .001, *d* = 2.39. Thus, by rejecting the independent model, we provide evidence for within-event dependency.[Fig-anchor fig1]

One possible issue with our design is that it requires the participant to engage in multiple retrieval trials. This procedure may artificially inflate the level of dependency. For example, retrieving elements B and C when cued by A may increase the associative strength between B and C, resulting in more consistent accuracy for all within-event elements in later retrieval trials. To address this possibility, we calculated dependency for the first retrieval trial of each event (as well as the second and third retrieval trial). We also calculated dependency for the independent model based on these individual trials to control for possible differences in accuracy across retrieval trials. Accuracy increased across retrieval trials (Trials 1–3: 51.9%, 58.2%, 62.3%), *F*(1.9, 22.7) = 9.53, *p* < .001, η_p_^2^ = .44. Despite this increase, no difference in dependency was seen across retrieval trials (Trials 1–3: 0.89, 0.86, 0.87), *F*(1.5, 17.6) = 0.98, *p* = .37, η_p_^2^ = .08. Indeed, when accounting for accuracy increases by subtracting dependency for the independent model from dependency for the data (data − independent model), a decrease in dependency was seen (Trials 1–3: 0.27, 0.21, 0.17), *F*(1.6, 19.9) = 6.39, *p* < .05, η_p_^2^ = .35. We could therefore find no evidence that testing events across multiple retrieval trials increased dependency. Instead, we saw decreases in dependency despite increased accuracy across retrieval trials.

## Experiment 2

Experiment 1 assessed dependency within three-element events using a cued-recall task. The observed dependency was greater than the Independent Model, providing evidence for episodic dependence. This result is in contrast to Trinkler et al. (2006) who, using a virtual reality encoding environment, failed to show any dependence for similar location-person-object triplets. One key difference between the studies is the use of a cued-recognition judgment in Trinkler et al. (2006) compared with the use of a cued-recall task here. In Experiment 2 therefore, we used a six-alternative forced-choice cued-recognition judgment. On a given retrieval trial, a single cue was presented (as in Experiment 1), and only one of the other elements was assessed (e.g., if cued by location, the person or the object was assessed). Six items (e.g., people) were presented on the screen beneath the cue (e.g., location) at the same time. Five of these items were associated with other learned events, and one item was associated with the cue item. The encoding conditions were identical to Experiment 1.

### Method

Experiment 2 was identical to Experiment 1 with the following exceptions:

#### Participants

Fifteen participants (11 men) gave informed consent to participate in Experiment 2. The mean age across participants was 24.3 years (*SD* = 5.0). By self-report, two participants were left-handed, and the remainder right-handed.

#### Materials

In order to equate overall performance across experiments, we increased the total number of episodes from 24 to 36. Stimuli were therefore 36 locations (e.g., a swimming pool), famous personalities (e.g., David Cameron), and common objects (e.g., a bicycle; see the Appendix).

#### Procedure

Use of 36 events created a total of 216 cued-recognition trials. All 36 events were tested across six blocks. Every event was tested with one of the cue-test pairs (e.g., cue: location, test: object) in each block, and for each block, each of the six cue-test pairs was used six times. Four test sequences were created and rotated across participants. For each test sequence, the order of cue-test pairs across blocks for each episode and the order of testing each event within each block were randomized.

On a given test trial, the cue and the six-alternative test items were presented simultaneously. The cue was presented in the middle of the screen with the six test items in two rows of three at the bottom of the screen. Participants were instructed to select the test item that was originally paired with the cue (e.g., which person was in the same event as this location?). They were required to respond within 5 s with a key press, and following this action were given a further 5 s to rate their confidence on a scale of 1–5. Participants were instructed to answer as accurately as possible within the time given.

### Results

#### Cued-recognition performance

Performance was well above chance (which would be 16.7% given the six-alternative forced-choice decision) with performance at 58% across cue type and retrieval type (see Table 2). Performance across the cue type and retrieval type appeared well matched, ranging from 56% to 61%. A one-way within-subject ANOVA across retrieval type (collapsed across cue type) revealed a significant effect, *F*(1.8, 25.3) = 3.51, *p* < .05, η_p_^2^ = .20, with greater accuracy when retrieving people, followed by locations and objects (note this is the opposite pattern to the trend seen in Experiment 1). No effect of cue type was seen, *F*(1.9, 26.3) = .46, *p* = .63, η_p_^2^ = .03. Across Experiments 1–2, we could therefore find no consistent differences in performance across locations, people, and objects. Finally, a one-way ANOVA revealed that cued-recognition performance differed across subjective confidence, *F*(2.6, 35.9) = 23.22, *p* < .001, η_p_^2^ = .62, with an increase in performance from lowest confidence (11.5% accuracy) to highest confidence (84.8% accuracy).

#### Dependency analysis

The mean proportion of trials in which both elements from an event were either successfully or unsuccessfully retrieved across participants (D) for each A_B_A_C_ and B_A_C_A_ analysis and each item type for the observed data are shown in Table 3. Note that in Experiment 1, the A_B_A_C_ analysis was a measure of within-trial dependency, as participants were required to retrieve both noncued items. In Experiment 2, the A_B_A_C_ analysis is a measure of between-trial dependency as a single retrieval trial tests only one of the noncued items.

A 2 × 3 (Analysis Type × Item Type) ANOVA on dependency of the observed data revealed a trend for a main effect of analysis type, *F*(1, 14) = 3.42, *p* = .09, η_p_^2^ = .20. The remaining main effect and interaction term did not approach significance, *F*s < .80, *p*s > .44, η_p_^2^s < .06. We could therefore find no consistent differences in dependency across analysis type and item type.

Dependency (D) for the data and independent model is shown in Figure 1. The data showed greater dependency than the independent model, *t*(14) = 3.50, *p* < .01, *d* = 0.91, in line with Experiment 1. Despite no difference in accuracy between Experiments 1 and 2, *t*(26) = 0.08, *p* = .94, *d* = 0.03, we found significantly less dependency in Experiment 2 than in Experiment 1, *t*(26) = 6.69, *p* < .001, *d* = 2.61. Note that we can make this direct comparison (i.e., without reference to the independent model) because accuracy was equated between Experiments 1 and 2. Thus, performance in the cued-recognition task showed greater dependence than the independent model, but less dependence than Experiment 1.

As in Experiment 1, we next investigated whether dependency changed across retrieval trials. As a single element was tested on each retrieval trial, we calculated dependency by pairing retrieval Trials 1–2, 3–4, and 5–6. As in Experiment 1, accuracy increased across retrieval trials (Pairs 1–3: 52.5%, 58.9%, 63.3%), *F*(1.6, 22.2) = 8.95, *p* < .01, η_p_^2^ = .39. Dependency also increased across retrieval trials (Pairs 1–3: 0.65, 0.74, 0.74), *F*(1.8, 25.3) = 5.49, *p* < .05, η_p_^2^ = .28. This increase in dependency was principally driven by the increase in accuracy however. Analysis of the difference between the data and the independent model (Trials 1–3: 0.06, 0.10, 0.04) revealed a significant effect, *F*(1.7, 23.3) = 4.29, *p* < .05, η_p_^2^ = .23, that was driven by a decrease in the difference between the independent model and the data between the second and third retrieval pair. Replicating Experiment 1, we therefore saw an increase in accuracy across retrieval trials but a decrease in dependency (once we accounted for increased accuracy).

In summary, Experiments 1 and 2 showed dependence greater than the independent model. Dependence however varied as a function of retrieval task, with greater dependence when memory was tested with a cued-recall (Experiment 1) than with a cued-recognition (Experiment 2) task. Finally, we could find no evidence in favor of increased levels of dependence across retrieval trials, suggesting our evidence for episodic dependency was not driven by repeated retrieval of events. Indeed, dependency decreased across retrieval trials in Experiments 1 and 2 despite increases in overall accuracy.

## Experiment 3

Given the finding that episodic dependency in Experiment 2 was significantly reduced relative to Experiment 1 by a change in retrieval task, we sought to both replicate and extend the findings of Experiments 1 and 2. In Experiment 3, we explored the difference between the cued recall used in Experiment 1 and the cued recognition used in Experiment 2 by having participants perform covert recall prior to performing cued recognition. Specifically, we were interested in seeing whether within-subject (inter-event) differences in dependency would reflect the subjective confidence of covert recall prior to the cued-recognition judgment.

Experiment 3 was identical to Experiment 2 except, at retrieval, participants were presented with the cue for 3 s and asked to “retrieve the entire episode.” They were then asked to judge how confident they were that they had retrieved one of the uncued elements (i.e., the element they were about to be tested on). Specifically, we instructed them to make this judgment as if it were a cued-recall trial (i.e., “How confident are you that you could tell the experimenter the person, when cued with the location?” as opposed to “How confident are you about getting the subsequent cued-recognition judgment correct?”).

Based on participants’ average subjective confidence for an event across the six retrieval trials for that event, we performed a median split into high confidence and low confidence events. Therefore, 18 events (with six cued-recognition trials per event) were labeled as low confidence and 18 events as high confidence. We designed this analysis to assess across-event differences in dependency based on overall confidence in retrieval trials relating to that event.

### Method

Experiment 3 was identical to Experiment 2 with the following exceptions:

#### Participants

Twenty-three participants (11 men) gave informed consent to participate in Experiment 3. The mean age across participants was 24.4 years (*SD* = 5.1). By self-report, all participants were right-handed.

#### Procedure

On a given test trial, a single cue was presented in the middle of the screen for 3 s. Participants were instructed to attempt to retrieve silently all information related to the event during this time (i.e., the two noncued items). They were then asked to judge how confident they were that they had correctly retrieved one of the noncued items (e.g., if cued by location, they were required to judge their confidence in retrieving the person). They responded with a key press on a scale of 1–4, with a further option below 1 of “no,” meaning they completely failed to retrieve the item. They were given 5 s to make this judgment. Subsequent to responding, participants carried out the same six-alternative forced-choice and confidence judgments as in Experiment 2, with the only difference being they were given 8 s to make the six-alternative judgment rather than 5 s.

### Results

#### Cued-recognition performance

As in Experiment 2, performance was well above chance, with performance across cue type and retrieval type at 71% (see Table 2). This higher performance relative to Experiment 2 (58%) is likely a result of the 3-s cue-only period where subjects were instructed to retrieve the entire episode. A three-way ANOVA across retrieval type failed to reveal a main effect, *F*(1.8, 40.7) = .40, *p* = .66, η_p_^2^ = .02. No significant effect was seen across cue type, *F*(1.8, 38.9) = 2.66, *p* = .09, η_P_^2^ = .11. Accuracy increased according to both prememory judgment confidence, *F*(3.1, 56.0) = 27.19, *p* < .001, η_p_^2^ = .60 (lowest–highest: 30.9%–91.2%), and postmemory judgment confidence, *F*(3.1, 56.1) = 27.26, *p* < .001, η_p_^2^ = .60 (lowest–highest: 30.8%–91.1%).

#### Performance for high versus low confidence events

Cued-recognition performance for low versus high confidence events are shown in Table 2. A 2 × 3 (confidence [high vs. low] × retrieval type [location vs. person vs. object]) ANOVA revealed a main effect of confidence, *F*(1, 22) = 45.74, *p* < .001, η_p_^2^ = .68, with higher accuracy for high- than low-confidence events. The main effect of retrieval type and the interaction failed to reach significance, *F*s < .27, *p*s > .75. A 2 × 3 ANOVA across cue type also revealed a main effect of confidence, *F*(1, 22) = 46.15, *p* < .001, η_p_^2^ = .68, with greater performance for high- than low-confidence events. The main effect of cue type and the interaction failed to reach significance, *F*s < 2.38, *p*s > .11. Memory performance was therefore higher for high-confidence than low-confidence events.

#### Dependency analysis

Prior to performing a median split of the events based on confidence, we collapsed across all events as in Experiments 1–2 (see Figure 1 and Table 3). A 2 × 3 (Analysis Type × Item Type) ANOVA on dependency of the observed data revealed main effects of analysis type, *F*(1, 22) = 28.70, *p* < .001, η_p_^2^ = .57, and item type, *F*(1.8, 38.8) = 4.72, *p* < .05, η_p_^2^ = .18, but no interaction, *F*(1.6, 35.9) = 2.00, *p* = .16, η_p_^2^ = .08. The main effect of analysis type showed greater dependency for the A_B_A_C_ than B_A_C_A_ analysis and the main effect of item type showed least dependency for objects (see Table 3). Note that these effects were not seen in Experiment 2 (though a trend for an effect of analysis type was present), suggesting they are not replicable across experiments. We therefore collapsed across these factors for comparison between the data and the independent model.

Dependency (D) for the data and independent model is shown in Figure 1. As in Experiments 1 and 2, the data showed greater dependency than the independent model, *t*(22) = 4.98, *p* < .001, *d* = 1.04. This difference in dependency (data − independent model: 0.04) was similar in magnitude to the difference in dependency seen in Experiment 2 (0.05), *t*(36) = 0.83, *p* = .41, *d* = 0.30, and significantly less than the difference seen in Experiment 1 (.20), *t*(34) = 11.83, *p* < .001, *d* = 3.97. Experiment 3 therefore replicated the decrease in dependency seen for cued-recognition relative to cued-recall tasks. Note that this occurs despite all three experiments having identical encoding instructions.

As in Experiment 2, we assessed dependency across Retrieval Trials 1–2, 3–4, and 5–6. Accuracy (Pairs 1–3: 0.69, 0.71, 0.74), *F*(1.9, 41.6) = 3.78, *p* < .05, η_p_^2^ = .15, and dependency (Pairs 1–3: 0.72, 0.77, 0.79), *F*(2.0, 40.1) = 6.94, *p* < .01, η_p_^2^ = .24, increased across retrieval pairs. Analysis of the difference between the data and the independent model (Pairs 1–3: 0.05, 0.06, 0.04) failed to reveal a significant effect, *F*(1.8, 30.3) = .92, *p* = .40, η_p_^2^ = .04. Therefore no difference in dependency was seen across retrieval trials once increases in overall accuracy were taken into account.

#### Dependency for high versus low confidence events

Splitting the events by prememory judgment confidence (average confidence across all six cued-recognition trials for each event; see Table 3 and Figure 1), we performed a 2 × 3 × 2 (Analysis Type × Item Type × Confidence) ANOVA on dependency of the observed data. This analysis showed there was a main effect of confidence, *F*(1, 22) = 40.25, *p* < .001, η_p_^2^ = .65, revealing greater dependency for high- than for low-confidence events (as expected given the higher accuracy for high-confidence events). Further main effects of analysis type, *F*(1, 22) = 28.66, *p* < .001, η_p_^2^ = .57, and item type, *F*(1.8, 38.7) = 4.48, *p* < .05, η_p_^2^ = .17, were also present (see earlier nonsplit analyses). Finally, interactions were seen between confidence and analysis type, *F*(1, 22) = 4.42, *p* < .05, η_p_^2^ = .17, and confidence and item type, *F*(2.0, 44.0) = 4.48, *p* < .05, η_p_^2^ = .17 (reflecting significant effects of analysis type and item type for low-confidence, *F*s > 9.77, *p*s < .001, but not high-confidence, *F*s < 1.8, *p*s > .19, events; see Table 3).

Collapsing across analysis type and item type, we compared low- and high-confidence events with the independent model. Although high-confidence events showed greater dependency than the independent model, *t*(22) = 2.74, *p* < .05, *d* = 0.60, no difference was seen for low-confidence events, *t*(22) = 0.85, *p* = .40, *d* = 0.16; the interaction term, however, failed to reach significance, *F*(1, 22) = 2.04, *p* = .17, η_P_^2^ = .09. These data suggest the level of dependency was therefore modulated by within-event confidence, with low confidence being associated with levels of dependency equivalent to the independent model.

## Modeling Episodic Dependency

### The Dependent Model

Across Experiments 1–3, we have provided evidence for episodic dependency. We consistently saw greater dependency than that predicted by the independent model. Indeed, the only situation where the independent model was able to account for the data was for low-confidence events in Experiment 3. In order to account for this dependency, we developed a dependent model, where within-event performance was modulated by an episodic factor (*E*^*i*^) that varies across events. This factor captures the extent to which the probability of correctly retrieving all the elements making up an event varies from the average probability across all events. When cued with A, this is estimated as the factor by which performance for that event (when cued with B or C) differs from the average performance across all events (when cued with B or C):

$$E^{i}=\;\left({T^{i}}_{BA}+{T^{i}}_{BC}+{T^{i}}_{CA}+{T^{i}}_{CB}\right)/(P_{BA}+P_{BC}+P_{CA}+P_{CB})\text{,}$$ 1

where for Event *i*, *T*^*i*^*_BA_* = 1 if the participant correctly retrieves A when cued by B, otherwise, *T*^*i*^*_BA_* = 0; that is, *T*^*i*^*_BA_* relates to the specific retrieval trial (*T*) for B when cued by A for Event *i*. Note that the episodic factor, for retrievals cued by A, does not include data from retrievals cued by A (i.e., *T*^*i*^_*AB*_, *T*^*i*^_*AC*_, *P*_*AB*_, *P*_*AC*_). As such, it is estimated using data from independent retrieval attempts. It should therefore capture variations in performance that have consistent effects across the multiple retrieval attempts concerning a given event. The probability of correctly retrieving a single item from a specific Event *i* is weighted by the episodic factor for that event. Thus, for the dependent model, for Event *i*, when cued by A, the probability of (a) correctly retrieving both B and C is equal to (*E*^*i*^)^2^*P_*AB*_*P*_*AC*_; (b) correctly retrieving B but not C is equal to *E*^*i*^*P*_AB_*(1 − *E*^*i*^*P*_*AC*_); (c) correctly retrieving C but not B is equal to (1 − *E*^*i*^*P*_*AB*_)*E^*i*^*P*_*AC*_, and (d) incorrectly retrieving B and C is equal to (1 − *E*^*i*^*P*_*AB*_)*(1 − *E*^*i*^*P*_*AC*_). Table 1 presents the contingency table for the dependent model across all *N* episodes. The independent model previously outlined corresponds to setting *E*^*i*^ = 1 across all events.

**Figure 2 fig2:**
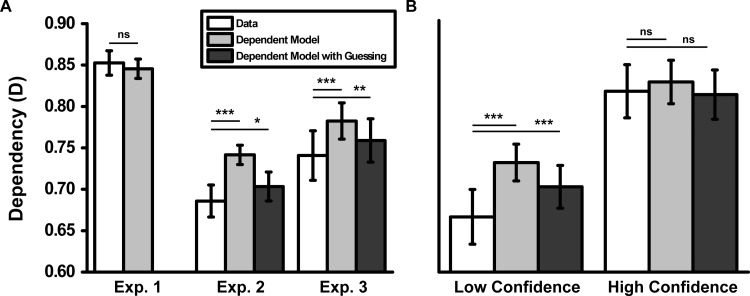
Mean dependency (D) for the observed data, the dependent model and the dependent model with guessing, collapsed across the A_B_A_C_ and B_A_C_A_ analyses and item type, for (A) Experiments 1–3 and (B) low and high confidence events for Experiment 3. Note that the dependent model with guessing is equivalent to the dependent model in Experiment 1 (hence, not shown), given that participants were explicitly instructed to answer “don’t know” instead of guessing. Therefore, the probability of participants answering correctly when they did not know was zero (i.e., *P*_G_/*c* = 0). Error bars represent ±1 standard error. Exp. = experiment; *ns* = nonsignificant. * *p* < .05. ** *p* < .01. *** *p* < .001.

For Experiment 1, we saw no significant difference between the data and the dependent model, *t*(12) = 1.20, *p* = .25, *d* = 0.30 (Figure 2). Thus, our dependent model seems to fully capture the level of dependency seen in cued recall. The dependent model overpredicted the level of dependency in Experiment 2, *t*(14) = 5.20, *p* < .001, *d* = 1.41, and Experiment 3, *t*(22) = 4.63, *p* < .001, *d* = 0.97 (when collapsed across high- and low-confidence events). The level of dependency during cued recognition was therefore greater than the independent model but less than the dependent model. These results serve to underlie how dependence differs as a function of retrieval task, with less dependency during cued recognition than cued recall.[Fig-anchor fig2]

Finally, whereas the dependent model overpredicted the level of dependency for low-confidence events in Experiment 3, *t*(22) = 5.18, *p* < .001, *d* = 1.07, no difference was seen between the data and the dependent model for high-confidence events, *t*(22) = 1.69, *p* = .11, *d* = 0.30. High-confidence events during cued recognition therefore appeared to show episodic dependency similar to that seen during cued recall (i.e., similar to the dependent model in both cases), whereas low-confidence events show reduced dependency (i.e., similar to the independent model). Our dependent model therefore does a good job in characterizing the level of dependence during cued recall and cued recognition for events where confidence is high. However, it consistently overpredicted the level of dependence for cued recognition (when not split by confidence and for low-confidence events).

One possible issue with our episodic factor is that although it does not include data from the retrieval trials actually being predicted (see Equation 1), it does include data from retrieval trials testing the same association (but in reverse). For example, if we are predicting retrieval performance for Element B when cued by A, we included performance for the retrieval trial testing A when cued by B. If the associative strength between these elements is symmetrical (i.e., the strength of the A–to–B association is related to the strength of the B–to–A connection; see Kahana, 2002), this might increase our ability to predict the level of dependency in our dependent model. To address this issue, we calculated *E*^*i*^ based on the retrieval trials for the association that was not of interest. For example, if we were interested in assessing the dependency between retrieval of Elements B and C when cued by A, we calculated *E*^*i*^ based on performance of the B–C association: *E*^*i*^ = (*C*^*i*^*_BC_* + *C*^*i*^*_CB_*)/(*P_BC_* + *P_CB_*). We compared this revised model with one where we calculated *E*^*i*^ based on performance for the retrieval of trials for the association of interest (but tested in reverse). In the previous example, this would be the B–to–A association and the C–to–A association: *E*^*i*^ = (*C*^*i*^_*BA*_ + *C*^*i*^_*CA*_)/(*P*_*BA*_ + *P*_*CA*_).

These revised models predicted equivalent dependency to each other for Experiment 1, *t*(12) < .001, *p* = .99, *d* = 0.05; Experiment 2, *t*(14) = 1.36, *p* = .20, *d* = 0.39; and Experiment 3, *t*(22) = 1.48, *p* = .15, *d* = 0.15. Therefore, building dependent models based on performance for the reverse of the associations of interest does not appear to predict the level of dependency more accurately than dependent models based on performance for the untested association. Thus, the inclusion of retrieval trials for the reverse of the associations of interest in the Dependent Model played no specific role in its ability to correctly predict the level of dependency in the data.

### The Dependent Model With Guessing

Across Experiments 1–3, we saw dependency differ as a function of retrieval task and event-specific retrieval confidence. Neither the independent model nor the dependent model was able to fully account for the pattern of dependency seen across experiments. What causes these differences in dependency? One possibility is that participants were more likely to guess in the forced-choice cued-recognition tests in Experiments 2–3 than during the cued-recall test in Experiment 1 (in which they were instructed to say “don’t know” rather than guess). Equally, participants may guess more for events associated with low versus high confidence. Guessing could render the dependent model inaccurate because some of the proportion of correct responses (which the dependent model assumes to be modulated by the episodic factor *E*^*i*^) actually arises from lucky guesses rather than from any memory process that would be modulated by an event-specific factor.

In Experiment 1, of the 43% of trials that were not correctly answered, 31% were “don’t know” responses and 12% were incorrect responses, and the chance of guessing correctly was low (1/24; i.e., if all the incorrect responses were guesses). We would therefore expect a maximum of 0.5% of responses in Experiment 1 to be lucky correct guesses. By contrast, in six-way forced-choice decisions (i.e., Experiment 2), the 42% of incorrect responses could all be guesses, which would imply around 8% of responses could be lucky correct guesses.

To take account of the proportion of guessed responses, we developed the dependent model with guessing. If guessing is occurring, the probability of retrieving B when cued by A (*P*_*AB*_) is the sum of the probability of “real” retrieval (*P*^*r*^_*AB*_) when not guessing (1 − *P*_*G*_) and the probability of guessing correctly (1/*c* in a *c*-way forced choice) when “real” retrieval fails (*P*_G_). In *c*–way forced choice, we have:

$$P_{AB}=(1-P_{G}){P^{r}}_{AB}+P_{G}/c.$$ 2

Now the dependent model is obtained by replacing *P*^*r*^_*AB*_ by *E*^*i*^*P*^*r*^_*AB*_, where *E*^*i*^ is calculated as before (Equation 1), and forming the contingency table from the adjusted estimate of *P*_*AB*_:

$$(1-P_{G})E^{i}{P^{r}}_{AB}+P_{G}\text{/}c=E^{i}{\text{(}P}_{AB}{-P}_{G}\text{/}c)+P_{G}\text{/}c\text{.}$$ 3

This gives the dependent model with guessing in Table 1, which reverts to the dependent model for *P*_*G*_ = 0, and reverts to the independent model for *E*^*i*^ = 1. Note that the independent model is unaffected by the proportion of guessed trials (*P*_*G*_) and that taking account of guessing effectively reduces the influence of factor *E*^*i*^ by removing guesses from the proportion of correct responses that it multiplies (see Equation 3).

Because we found similar performance across cue type and retrieval type, we assumed a single proportion of guessed trials (*P*_*G*_) for each subject, irrespective of cue type and retrieval type. The level of guessing is unknown but is constrained by the fact that the proportion of errors caused by guessing, (*c* − 1)*P*_*G*_/*c*, is no greater than the overall proportion of errors, giving:

$$P_{G}\leq [1-\left(P_{AB}+P_{BA}+P_{BC}+P_{BC}+P_{AC}+P_{CA}\right)/6]c/(c-1)\text{.}$$ 4

The dependent model with guessing, assuming that all errors reflect guessing (i.e., assuming equality in Equation 4), provides a good match to the level of dependency in Experiments 2 and 3 (see Figure 2). Though we saw significantly less dependency for the data than the dependent model with guessing for Experiment 2, *t*(14) = 2.46, *p* < .05, *d* = 0.62, and Experiment 3, *t*(22) = 3.01, *p* < .01, *d* = 0.67, the effect sizes were small relative to those of the dependent model (Experiment 2, *d* = 1.41; Experiment 3, *d* = 0.97) and the independent model (Experiment 2, *d* = 0.91; Experiment 3, *d* = 1.05). Finally, we saw no difference between the data and the dependent model with guessing for high-confidence events, *t*(22) = 0.47, *p* = .65, *d* = 0.17, but a significant difference was seen for low-confidence events, *t*(22) = 4.22, *p* < .001, *d* = 0.86 (though again the effect size was small relative to the dependent model, *d* = 1.07).

The dependent model with guessing therefore provides a good fit to the data across Experiments 2 and 3, suggesting the decrease in dependency during cued recognition compared with cued recall was at least partly driven by differences in the level of guessing between retrieval tasks (with increases in guessing decreasing dependency). Although the dependent model with guessing cannot fully accommodate all the results of Experiment 3, it appears to explain a large proportion of variance in both Experiments 2 and 3 (mean 91.4% of between-subjects variance across Experiments 2–3, relative to 84.9% for the independent model and 89.0% for the dependent model). Therefore, we have provided evidence that the retrieval of events is modulated by a within-event episodic factor that varies across events (once changes in the level of guessing across retrieval task and confidence are taken into account).

## General Discussion

Long-standing neuroscientific and psychological theories of memory predict that the multimodal elements of an episodic event are bound together within the hippocampus. We have introduced a novel method to assess this theorized binding. By building independent and dependent models, based on a participant’s individual behavior, we were able to assess episodic dependency while controlling for overall memory performance. Across three experiments, we were consistently able to reject the independent model, demonstrating interdependence in the ability to retrieve the different elements comprising the same event. This episodic dependency was seen both within retrieval trials requiring recall of two elements (Experiment 1) and across multiple retrieval trials concerning individual elements from the same event (all experiments). The dependency seen across multiple retrieval trials concerning elements from the same event indicates that retrieval of an event reflects a common factor that varies across events rather than across retrieval trials.

Though we consistently found evidence for episodic dependency, the degree of dependency was shown to vary as a function of retrieval task and the average subjective confidence of covert recall prior to (multiple) retrievals of a given event. This indicates that the nature of the retrieval task, as well as an individual’s subjective confidence concerning an event, is relevant for our analyses. These differences appeared to be partially driven by differences in the level of guessing across retrieval task and confidence. We discuss the functional implications of these findings in the following text.

### The Cause of Within-Event Dependency

We observed greater dependence between the retrieval of different elements of the same event than predicted by the average performance level across all retrieval trials (i.e., more dependence than the independent model). This increased dependence was observed across multiple retrieval trials (three trials in Experiment 1; six trials in Experiments 2–3). Thus, the variation in performance across all retrieval trials in an experiment tended to reflect variation *between events*—performance on retrieving the individual elements composing a given event is similar (i.e., the ability to retrieve one element tends to be predicted by the average ability to retrieve the other elements in the same event: factor *E*^*i*^ in the dependent model). Confirming this, analysis of the raw response data (i.e., correct or incorrect on each trial) revealed that between-event variation accounted for ∼27% of the total variance.

What potential mechanisms might explain these results? For simplicity, we suppose that an event is encoded as pairwise associations among its constituent elements. Then one model of retrieval would be that activation of the cue item causes reactivation of the retrieved item via the corresponding association, with performance reflecting associative strength. In this model, a between-events modulation of encoding strength, perhaps due to variations in general attention or arousal, would produce dependence between the retrieval of the elements of the same event, because they reflect the same modulatory factor (factor *E*^*i*^ in the dependent model).

In an alternative model, the cued retrieval of one element of an event might reflect a process of pattern completion in which all elements are re-activated via all of the within-event associations. In this case, retrieval of each element of an event would reflect the average strength of all pairwise associations within that event, thus creating an interdependence that would also be modeled by the factor *E*^*i*^ in the dependent model, even though the strengths of the association themselves were independent.

The current data do not distinguish between the two alternative models outlined above. Further experiments will be necessary. For example, presenting elements of an event in a pairwise manner across encoding trials (in an analagous manner to Zeithamova, Dominick, & Preston, 2012) would presumably not allow for a common encoding factor (such as attention) to mediate the associative strength between all within-even elements. The presence of episodic dependency under these encoding conditions would therefore favor the pattern completion rather than the common associative strength account. Alternatively, increasing the number of elements within each event should increase reaction times during cued retrieval of a single element according to the pattern-completion explanation, but not under the common modulation of associative strength explanation.

### Retrieval-Related Factors Affecting Within-Event Dependency, and Guessing

Although we consistently rejected the independent model across three experiments, we also found that the level of within-event dependency varied significantly with retrieval task. Whereas in Experiment 1 (using a cued-recall task) showed high levels of dependency, Experiment 2 (using a cued-recognition task) showed lower levels of dependency. How does the retrieval task modulate dependency? We introduced a dependent model with guessing, given that participants may have relied more heavily on guessing during cued recognition than cued recall and that the episodic factor (*E*^*i*^) in the dependent model should not be applied to the proportion of correct responses that arise from guessing (see Equations 2 and 3). Once we incorporated guessing into the dependent model, we were able to explain a large proportion of the variance of Experiment 2. These results suggest that cued recognition may rely on retrieval mechanisms similar to cued recall and show similar levels of dependence, once the increased guessing in cued recognition is taken into account. Despite this, the dependent model with guessing was not fully able to explain the results of Experiments 2 and 3. If future experiments reveal a consistent overprediction in dependency for cued recognition, it may relate to an underlying difference with cued recall, such as the extent to which pattern completion is required at retrieval.

To extend these results, in Experiment 3, we investigated whether differences in dependency could be seen between events (within-subject) according to whether they were rated with low or high subjective confidence following covert recall (averaged over the retrieval trials concerning that event). We showed greater dependency for high- than for low-confidence events. Low-confidence events were presumably associated with greater levels of guessing and therefore less dependency. Again, incorporating guessing into our dependent model provided a better fit to the data than the dependent model. This result suggests that the main difference between high- and low-confidence events is the level of guessing when answering questions concerning that event (i.e., across multiple retrieval trials), with similar levels of dependency seen once this is taken into account.

Despite this, dependency for low-confidence events showed less dependence than the dependent model with guessing. If this deficit in dependence is seen consistently over future experiments, it is possible that low-confidence events are encoded in a less holistic way than high-confidence events. Perhaps some pairwise associations within low-confidence events are so weak as to fail to benefit from between-event modulation at encoding or to fail to contribute to pattern completion at retrieval.

Finally, we note that the 3-s covert recall period between presentation of the cue and the six-alternative response options in Experiment 3 improved memory performance relative to Experiment 2. Thus, accuracy improves when participants are encouraged to covertly recall the entire event prior to a cued-recognition judgment. This improvement in performance was not, however, associated with increased dependency. Indeed, the dependent model with guessing provided a good fit to both Experiments 2 and 3. Participants were able to use the 3-s cue-only period of Experiment 3 to increase the probability of successfully retrieving the correct event element, but this procedure, when successful, appears to have simply increased confidence and performance and reduced guessing without otherwise changing the amount of within-event dependence.

### Implications for Models of Hippocampal Episodic Binding

The cued recall and cued recognition tasks used here are examples of the *episodic*, *associative*, *declarative*, or *relational* memory thought to depend on the hippocampus (Cohen & Eichenbaum, 1993; Kinsbourne & Wood, 1975; O’Keefe & Nadel, 1978; Scoville & Milner, 1957; Squire & Zola-Morgan, 1991), and a similar object–person–place memory task was previously shown to be impaired by hippocampal damage (Spiers, Burgess, Hartley, et al., 2001; Spiers, Burgess, Maguire, et al., 2001). Although nonhippocampal medial temporal lobe regions, such as the perirhinal cortex, have been implicated in unimodal associative processes (Mayes et al., 2004, 2007) or complex conjunctive representations of objects (Barense, Gaffan, & Graham, 2007; Bussey & Saksida, 2007), such regions are not thought to support associations between multimodal representations. Indeed, in a recent study, Horner et al. (2012) found evidence for a double dissociation between these regions, with nonhippocampal medial temporal lobe regions selectively contributing to recognition memory for single items (words) and the hippocampus selectively contributing to the successful retrieval of (word–scene) pairwise associations.

The imagery task and memoranda used here were designed to require cross-modal binding, given that the hippocampus is thought to act as a convergence zone, binding multimodal information represented in distinct neocortical regions into event engrams (Damasio, 1989; Marr, 1971). In particular, the relatively high proportion of recurrent collaterals in region CA3 of the hippocampus has been associated with the ability to bind such multimodal information, via the *collateral effect* (Marr, 1971) or *pattern completion* (Gardner-Medwin, 1976; Hopfield, 1982; McClelland et al., 1995; Treves & Rolls, 1992). Thus, both the anatomical situation of the hippocampus, at the top of a hierarchy of inputs from neocortical regions, and the architecture of CA3 provide the ideal platform for rapidly binding elements of an event into a coherent representation.

We analyzed our results in terms of a model of memory in which each event is encoded by pairwise associations between its constituent elements. This is consistent with the anatomy (see earlier discussion) and with the observation that single neurons within the hippocampus fire in response to specific environmental locations in both rodents (O’Keefe & Dostrovsky, 1971) and humans (Ekstrom et al., 2003), and in response to specific famous people in humans (Quiroga, Reddy, Kreiman, Koch, & Fried, 2005). We presume neurons in the hippocampus representing distinct elements initially increase their associative strength due to the co-occurrence of two elements. Over time, however, such cells may come to activate equally for both elements, effectively coding a combined two-element representation (Miyashita, 1993; Schapiro, Kustner, & Turk-Browne, 2012).

We interpreted the dependence we observed between retrieval of different elements of the same event in terms of two types of model. Under one type of model, the strength of pairwise associations relating to the same event is uniformly affected by an encoding factor that varies between events. At retrieval, the probability of retrieving different elements from the same event is related because the strengths of the corresponding associations are all modulated by the same factor (*E*^*i*^ in Equation 1). Thus, if attention is high for the encoding of a specific event, stronger pairwise associations might be formed, possibly via increased firing rates and thus increased long-term potentiation, as hypothesized for place cell firing (e.g., Fenton et al., 2010; Kentros, Agnihotri, Streater, Hawkins, & Kandel, 2004).

Under the alternative type of model, all cued-retrieval trials rely on a process of pattern completion in which activation from neurons representing the cue element reactivates neurons representing all of the other elements via all of the pairwise associations between them. Thus, all within-event pairwise associations contribute to the final retrieval state, regardless of the element that is actually required to be retrieved, and the ability to retrieve all elements from the event are related to mean performance by the same overall factor (again, *E*^*i*^ in Equation 1). This model is consistent with the collateral effect (Marr, 1971) or pattern-completion effect simulated in many models of the hippocampus as an associative memory network (Gardner-Medwin, 1976; Hopfield, 1982; McClelland et al., 1995; Treves & Rolls, 1992). Direct evidence for pattern completion in the hippocampus can be seen in the way that place cell firing evolves when a rat is placed in an environment intermediate to two distinct, familiar environments (Wills, Lever, Cacucci, Burgess, & O’Keefe, 2005) or in the way that place cell firing is robust to removal of subsets of environmental cues unless NMDA receptors in CA3 are knocked out (Nakazawa et al., 2002). Future experiments will be required to distinguish between these two types of models, as discussed earlier.

Finally, the two types of abstract model we outlined are consistent with several different implementations. In addition to the direct implementation of encoding factors or pattern completion at retrieval discussed previously, our evidence for episodic dependency accords with context models of episodic memory, whereby items (elements) seen at encoding are bound to a time-varying representation of context (Estes, 1955; Howard & Kahana, 2002). In particular, the temporal context model (Howard & Kahana, 2002; Polyn & Kahana, 2008) would predict that the 3 elements present at encoding of a single event would be bound to a contextual representation (and contribute to that representation). At retrieval the presentation of a single element results in the retrieval of the associated context, including information relating to the two noncued elements. As such, retrieval of the contextual representation itself could introduce shared variance at encoding and could also support pattern completion at retrieval.

### Potential Methodological Issues

Our dependency analyses necessarily required assessing memory for a single event across multiple retrieval trials. These multiple retrieval trials might have served to increase the associative strength within-event, increasing the likelihood of seeing episodic dependency. However, we could find no evidence for such a mechanism; dependency did not increase across multiple retrieval trials once we had controlled for increases in accuracy. Our main conclusions would have held regardless of whether we analyzed the first or last retrieval trial.

Despite increases in accuracy, Experiments 1 and 2 (but not 3) showed decreases in dependency across retrieval trials. One possible explanation for such a decrease in dependency (at least in Experiment 2) would be retrieval-induced forgetting (Anderson et al., 1994). Here the repeated selective retrieval of a single within-event element (e.g., B when cued by A) leads to the inhibition of the other nontested element (e.g., C). Such an inhibitory process could increase the difference in strength between within-event associations, resulting in decreased dependency. We did not see such a decrease in Experiment 3, however, suggesting that if such a process were present, it is not reliable within the present experimental paradigm.

A further potential explanation for dependency for two elements (e.g., person and object) when cued by a single element (e.g., location) would be a “good cue” effect. If some locations acted as a better cue than others, then the probability of retrieving the other two within-event elements would be increased for that location and decreased for other locations, resulting in dependency. However, we also showed dependency in our analysis of retrieval of a single element (e.g., location) when cued by the other two elements (e.g., person and object) across separate retrieval trials. Dependency in this analysis could not have resulted from a single good cue effect (though we note that dependency was reduced for this analysis in Experiment 3). In addition, there were no systematic differences in dependence across the different cue types and retrieval types, ruling out that dependence reflects variations in the memorability of a specific type of element.

Finally, we constructed three separate models (the independent, dependent and dependent model with guessing) in an attempt to predict the level of dependency seen in the data across three experiments. These models had no “free parameters” that were allowed to vary in order to minimize the difference between the data and the model. For example, the episodic factor *E*^*i*^ was calculated on the basis of the ratio of performance for a single event relative to performance across all events. There is no specific reason why inclusion of *E*^*i*^ would allow for a better model fit than if *E*^*i*^ were set to 1 (as in the independent model). Indeed, we saw situations, such as for low-confidence events in Experiment 3, where the independent model fit the data better than the dependent model.

### Summary

We have presented three experiments that provide consistent evidence for episodic dependency. This evidence supports the assumption, made by many models of the hippocampus and episodic memory, that the multimodal information that forms an event is bound within a coherent associative structure or event engram, such that the ability to retrieve different elements of the same event is related. These results are readily explicable by means of a dependent model (including guessing) in which a single episodic factor, that varies across events, uniformly affects the retrieval of the various elements comprising the same event. This factor might reflect a common (e.g., attentional) modulation of the strength of encoding of within-event associations or a process of pattern completion by which all within-event associations contribute to the cued retrieval of each element. Both potential mechanisms are consistent with several aspects of the neurophysiology of the hippocampus. The specific mechanism responsible for episodic dependency will require further experiments to identify. The procedure provided here will allow quantitative analysis of this within-event dependency, which is an important feature of episodic memory and one that many models assume to be the characteristic contribution of the hippocampus.

## Figures and Tables

**Table 1 tbl1:** Contingency Table for the Independent Model, Dependent Model, and Dependent Model With Guessing, for Successful Retrieval of Elements B and C When Cued With Element A

	Retrieval of Element B	
Retrieval of Element C	Correct (*P*_*AB*_)	Incorrect (1 − *P*_*AB*_)
Independent model		
Correct (*P*_*AC*_)	*P*_*AB*_*P*_*AC*_	*P*_*AC*_(1 − *P*_*AB*_)
Incorrect (1 − *P*_*AC*_)	*P*_*AB*_ (1 − *P*_*AC*_)	(1 − *P*_*AB*_)(1 − *P*_*AC*_)
Dependent model		
Correct (*P*_*AC*_)	*P*_*AB*_*P*_*AC*_ ∑_1_^*N*^(*E*^*i*^)^2^	*P*_*AC*_ ∑_1_^*N*^ *E*^*i*^(1 − *E*^*i*^*P*_*AB*_)
Incorrect (1 − *P*_*AC*_)	*P*_*AB*_ ∑_1_^*N*^ *E*^*i*^(1 − *E*^*i*^*P*_*AC*_)	∑_1_^*N*^ (1 − *E*^*i*^*P*_*AB*_)(1 − *E*^*i*^*P*_*AC*_)
Dependent model with guessing		
Correct (*P*_*AC*_)	∑_1_^*N*^[*E*^*i*^(*P*_*AB*_ − *P*_*G*_*/c*) + *P*_*G*_*/c*][*E*^*i*^(*P*_*AC*_ − *P*_*G*_*/c*) + *P*_*G*_*/c*]	∑_1_^*N*^[1 − {*E*^*i*^(*P*_*AB*_ − *P*_*G*_/*c*) + *P*_*G*_*/c*}][*E*^*i*^(*P*_*AC*_ − *P*_*G*_/*c*) + *P*_*G*_/*c*]
Incorrect (1 − *P*_*AC*_)	∑_1_^*N*^[*E*^*i*^(*P*_*AB*_ − *P*_*G*_*/c*) + *P*_*G*_*/c*][1 − {*E*^*i*^(*P*_*AC*_ − P_*G*_*/c*) + *P*_*G*_*/c*}]	∑_1_^*N*^[1 − {*E*^*i*^(*P*_*AB*_ − *P*_*G*_/*c*) + *P*_*G*_*/c*}][1 − {*E*^*i*^(*P*_*AC*_ − *P*_*G*_/*c*) + *P*_*G*_*/c*}]
*Note*. Dependent model equates to the independent model if *E*^*i*^ (episodic factor) = 1. Dependent model with guessing equates to the dependent model if *P*_G_ (probability when guessing) = 0 and equates to the independent model if *E*^*i*^ = 1. The table gives the proportion of responses falling into the four categories.		

**Table 2 tbl2:** Percentage of Correct Cued Recall (for Experiment 1), Cued Recognition (for Experiments 2–3), and Low/High Confidence Across Events (for Experiment 3) for Each Retrieval and Cue Type

	Retrieval type		
Cue type	Location	Person	Object
Experiment 1			
Location	n/a	55.8 (29.4)	57.7 (25.8)
Person	56.1 (30.2)	n/a	61.2 (27.4)
Object	59.0 (27.4)	55.1 (29.8)	n/a
Experiment 2			
Location	n/a	59.8 (28.8)	54.6 (27.3)
Person	58.7 (25.4)	n/a	59.1 (22.8)
Object	55.6 (28.0)	61.5 (25.4)	n/a
Experiment 3			
Location	n/a	70.2 (25.1)	68.7 (27.9)
Person	71.0 (28.3)	n/a	72.7 (24.7)
Object	71.1 (25.3)	73.7 (21.4)	n/a
Low confidence			
Location	n/a	59.9 (27.4)	59.2 (29.4)
Person	61.4 (32.5)	n/a	64.0 (28.0)
Object	63.8 (26.7)	66.2 (23.3)	n/a
High confidence			
Location	n/a	80.4 (24.7)	78.3 (29.0)
Person	80.7 (26.8)	n/a	81.4 (23.6)
Object	78.5 (27.2)	81.2 (22.2)	n/a
*Note*. Retrieval type refers to the element the participants were tested on, and cue type refers to the element the participants were cued with. Standard deviations appear in parentheses. n/a = not applicable.			

**Table 3 tbl3:** Mean Dependency (and Standard Deviation) for the A_B_A_C_ and B_A_C_A_ Analyses for the Observed Data for Experiments 1–3 and Low/High Confidence Across Events for Experiment 3

	A_B_A_C_ analysis			B_A_C_A_ analysis		
Experiment	Location cue—Person/object retrieval	Person cue—Location/object retrieval	Object cue—Location/person retrieval	Object/person cue—Location retrieval	Location/object cue—Person retrieval	Location/person cue—Object retrieval
Experiment 1	0.89 (0.07)	0.89 (0.07)	0.84 (0.08)	0.83 (0.10)	0.86 (0.06)	0.80 (0.11)
Experiment 2	0.69 (0.09)	0.71 (0.10)	0.70 (0.10)	0.68 (0.12)	0.69 (0.10)	0.65 (0.10)
Experiment 3	0.77 (0.14)	0.76 (0.16)	0.73 (0.15)	0.75 (0.14)	0.70 (0.15)	0.72 (0.17)
Low confidence	0.73 (0.14)	0.69 (0.18)	0.65 (0.18)	0.68 (0.16)	0.60 (0.18)	0.64 (0.18)
High confidence	0.81 (0.16)	0.83 (0.17)	0.82 (0.14)	0.82 (0.16)	0.80 (0.17)	0.80 (0.21)

## Full List of 36 Locations, People, and Objects Used in Experiments 2 and 3

**Table:**

Locations	People	Objects
Train station	George Clooney	Football
Supermarket	Britney Spears	Wallet
Cinema	Prince William	Hammer
Restaurant	Beyonce	Light bulb
Pub	Justin Timberlake	Battery
Office	Hilary Clinton	Skateboard
Car park	David Cameron	Necklace
Gym	Angelina Jolie	Violin
Bowling alley	George Bush	Pencil case
Lift	Tony Blair	Handbag
Tree house	Barack Obama	Pram
Living room	David Beckham	Trophy
Hospital	Jennifer Lopez	Spade
Church	Robert De Niro	Bike
Swimming pool	Mick Jagger	Calculator
Cruise ship	Gordon Brown	Carrot
Coffee shop	Wayne Rooney	Camera
Zoo	Stephen Hawking	Book
Nightclub	Sean Connery	Trumpet
Park	Johnny Depp	Basket
School yard	Kylie Minogue	Umbrella
Police station	Margaret Thatcher	Mug
Beach	Lady Gaga	Television
Stadium	Kate Middleton	Cricket bat
Ski lift	Bill Gates	Magnet
Kitchen	Tom Cruise	Mirror
Bedroom	Madonna	Whisk
Basement	Clint Eastwood	Paintbrush
Patio	Paul McCartney	Jug
Airport	Harrison Ford	Screwdriver
Motorway	Oprah Winfrey	Chainsaw
River	John Travolta	Sewing machine
Cornfield	Julia Roberts	Sleeping bag
Bank	Pamela Anderson	Toothbrush
Hair salon	Meryl Streep	Dice
Casino	Angela Merkel	Binoculars
*Note*. In Experiment 1, a subset of 24 was used.		
